# Structural Basis for the Acceleration of Procollagen Processing by Procollagen C-Proteinase Enhancer-1

**DOI:** 10.1016/j.str.2018.06.011

**Published:** 2018-10-02

**Authors:** David Pulido, Urvashi Sharma, Sandrine Vadon-Le Goff, Sadaf-Ahmahni Hussain, Sarah Cordes, Natacha Mariano, Emmanuel Bettler, Catherine Moali, Nushin Aghajari, Erhard Hohenester, David J.S. Hulmes

**Affiliations:** 1 Department of Life Sciences, Imperial College, London SW7 2AZ, UK; 2 UMR5086, CNRS/Université Claude Bernard Lyon 1, 69367 Lyon Cedex 7, France; 3 UMR5305, CNRS/Université Claude Bernard Lyon 1, 69367 Lyon Cedex 7, France

**Keywords:** extracellular matrix, collagen, fibrosis, metalloproteinase, macromolecular complex

## Abstract

Procollagen C-proteinase enhancer-1 (PCPE-1) is a secreted protein that specifically accelerates proteolytic release of the C-propeptides from fibrillar procollagens, a crucial step in fibril assembly. As such, it is a potential therapeutic target to improve tissue repair and prevent fibrosis, a major cause of mortality worldwide. Here we present the crystal structure of the active CUB1CUB2 fragment of PCPE-1 bound to the C-propeptide trimer of procollagen III (CPIII). This shows that the two CUB domains bind to two different chains of CPIII and that the N-terminal region of one CPIII chain, close to the proteolytic cleavage site, lies in the cleft between CUB1 and CUB2. This suggests that enhancing activity involves unraveling of this chain from the rest of the trimer, thus facilitating the action of the proteinase involved. Support for this hypothesis comes from site-directed mutagenesis, enzyme assays, binding studies, and molecular modeling.

## Highlights

• The crystal structure of PCPE-1 bound to the C-propeptides has been determined
• The N terminus of one propeptide chain binds to the CUB1CUB2 fragment of PCPE-1
• PCPE-1 seems to unravel the propeptide trimer to enable proteolytic release
• Molecular modeling with the proteinase and its substrate supports this hypothesis

## Graphical Abstract

**Figure:**
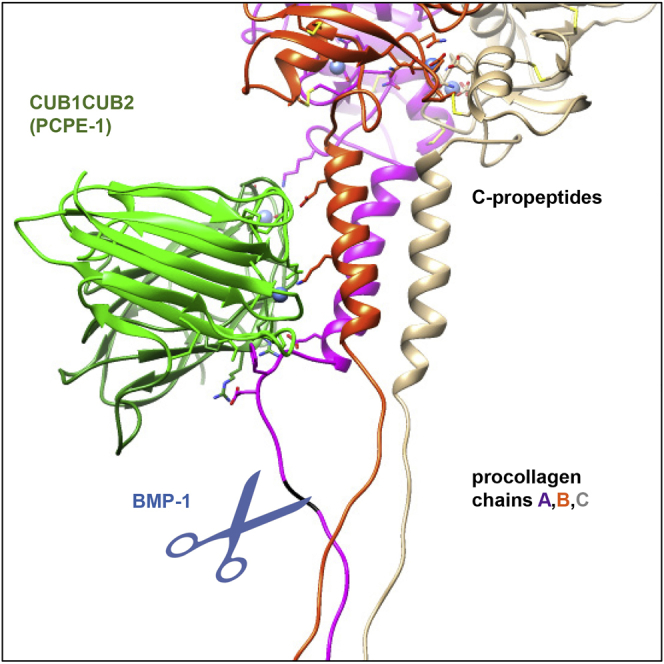

## Teaser

Collagens are the most abundant proteins in mammals. Fiber-forming collagens are synthesized as procollagens, where the C-propeptides are released by specific proteinases (BMP-1). C-propeptide release is accelerated by another extracellular matrix protein called PCPE-1. Here, based on structural data, the authors present a mechanism for the acceleration of C-propeptide release.

## Introduction

Fibrosis, an important pathogenic factor in diseases affecting most organs (notably heart, liver, and lung), contributes to up to 45% of deaths in the developed world (Rockey et al., 2015, Mehal et al., 2011). It is characterized by excessive accumulation of extracellular matrix (ECM), in response to injury, infection, and inflammation, mainly in the form of collagen fibers. Despite the importance of fibrosis, currently there are no approved antifibrotic therapies. One approach is to control the deposition of collagen fibers by targeting their biosynthetic pathway. Among the 28 different genetic types of collagen in humans (Ricard-Blum, 2011), the so-called fibrillar collagens (types I, II, III, V, and XI) are synthesized in precursor form, procollagens (∼450 kDa), where the central triple-helical region is flanked by trimeric N- and C-terminal globular propeptides (∼50 kDa and ∼90 kDa, respectively). These are removed by specific proteinases, usually the bone morphogenetic protein-1 (BMP-1)/tolloid-like metalloproteinases (BTPs) for the C-propeptides (Vadon-Le Goff et al., 2015). The BTPs consist of an N-terminal astacin-like catalytic domain followed by several CUB (complement C1r/C1s, Uegf, Bmp-1) and EGF (epidermal growth factor) domains. which are important for substrate specificity (Wermter et al., 2007, Berry et al., 2009). The rate-limiting step in the assembly of mature collagen molecules into fibrils is the release of the C-propeptides (Kadler et al., 1987). In addition to fibrillar procollagens, BTPs act on other ECM substrates as well as growth factor precursors or antagonists, raising the possibility of off-target effects when using BTP inhibitors to control C-propeptide cleavage (Vadon-Le Goff et al., 2015).

Some time ago Adar et al. (1986) described an ECM protein that accelerates the release of the C-propeptides from procollagen I by BMP-1 by up to 20-fold, called procollagen C-proteinase enhancer-1 (PCPE-1). This protein (∼55 kDa) consists of two CUB domains and a C-terminal NTR domain, where the intact CUB1CUB2 region (henceforth C1C2) is both necessary and sufficient for enhancing activity (Kronenberg et al., 2009). Previous studies have revealed that the role of PCPE-1 is specific for release of the C-propeptides from fibrillar procollagens, having no effect on BMP-1 activity for a range of other substrates (Moali et al., 2005, Petropoulou et al., 2005). This observation raised the possibility that PCPE-1 might be an attractive target for controlling BTP activity and hence the deposition of collagen fibrils in the ECM.

Early observations showed that maximum enhancing activity occurs when the amounts of PCPE-1 and procollagen are approximately equivalent, suggesting that PCPE-1 forms a complex with the substrate (Moschcovich et al., 2001). This was later confirmed and the binding site localized to the C-propeptide region, with a dissociation constant in the nanomolar range (Vadon-Le Goff et al., 2011). Further studies by small-angle X-ray scattering (SAXS) revealed that C1C2 binds to the stalk/base region of the free C-propeptide trimer, which has the shape of a flower (Bourhis et al., 2012a, Sharma et al., 2017), close to the site where it is cleaved from the rest of the procollagen molecule by BTPs (Bourhis et al., 2013). Also, kinetic studies have shown that saturating concentrations of PCPE-1, or C1C2, increase the catalytic efficiency (*k*_cat_/*K*_M_) of BMP-1 by over 10-fold, due to a small reduction in *K*_M_ and a ∼ 4-fold increase in *k*_cat_, using either procollagen I (Moschcovich and Kessler, 2016) or a shortened form of procollagen III (Bourhis et al., 2013). Despite these results, two questions remain. Why is the binding stoichiometry one molecule of PCPE-1 (or C1C2) to one C-propeptide trimer? And more generally, what is the molecular mechanism by which PCPE-1 binding enhances the activity of BTPs? Here we present the crystal structures of two forms of a complex between the active C1C2 region of PCPE-1 and the C-propeptide trimer of procollagen III. Together with site-directed mutagenesis, enzyme assays, binding studies, and molecular modeling, these results not only account for the observed stoichiometry but also provide a plausible mechanism for enhancement through localized unraveling of the procollagen trimer.

## Results

**Figure 1:**
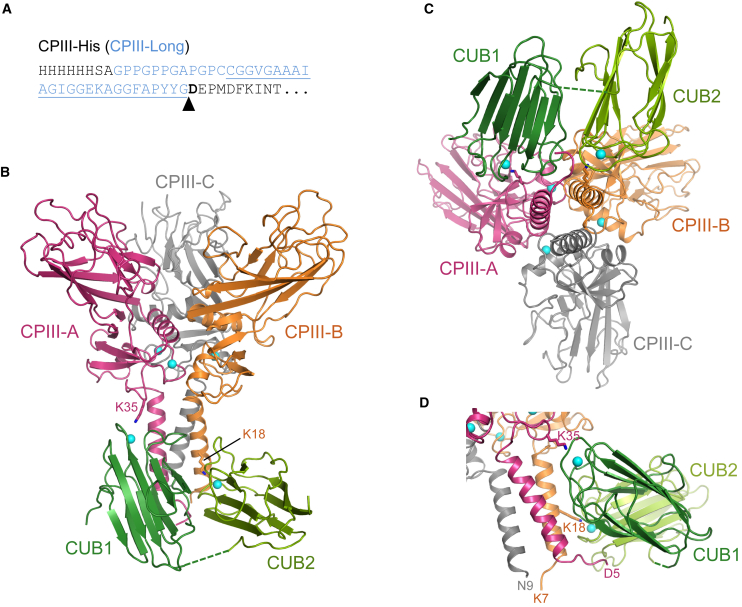
Overall Structure of the CPIII:C1C2 Complex (A) N-terminal amino acid sequences of CPIII-His and CPIII-Long. The additional sequence shown in blue, present only in CPIII-Long, contains the C-telopeptide region (underlined). The BTP cleavage site is indicated with an arrow and residue Asp1 at the P1′ position is in bold. (B) Side view showing C1C2 attached to the stalk/base region of CPIII. The individual chains of CPIII (A, B, and C) are in magenta, orange, and gray, respectively, and the CUB1 and CUB2 domains of PCPE-1 in dark green and olive green, respectively. Ca^2+^ ions are the cyan spheres. The linker connecting CUB1 and CUB2 (absent in the electron density map) is shown as a dotted line. (C) View down the axis of the coiled coil (from the N terminus) showing CUB1 and CUB2 bound to chains A and B of CPIII. (D) Side view showing the N-terminal region of CPIII chain A interacting with C1C2. See also Figure S1.

Two forms of the complex were crystallized using the active C1C2 fragment of human PCPE-1 (Bourhis et al., 2013). For the first, C1C2 was complexed with the C-propeptide trimer of procollagen III (CPIII-His) where each chain consists of an N-terminal His tag followed by the entire sequence of the C-propeptide (245 residues) starting at Asp1 at the P1′ position of the BMP-1 cleavage site (Figure 1A). For the second we used CPIII-Long, which includes a 37-residue insert between the His tag and the C-propeptide of CPIII-His (Figure 1A). This insert consists of the last three Gly-X-Y triplets of the triple helix and the entire C-telopeptide sequence N-terminal to the BMP-1 cleavage site (Bourhis et al., 2013). For both CPIII-His and CPIII-Long, residue numbering begins at Asp1 (Figure 1A) with negative numbers used for more N-terminal residues. Crystal structures were determined to resolutions of 2.70 Å and 2.78 Å, respectively (Table 1). In the structure of CPIII-His:C1C2, C1C2 is complete for residues 8–250 (numbered from the N-terminal residue following removal of the signal peptide) except for residues 126–132 corresponding to the inter-domain linker. Each chain of CPIII-His is complete to the C terminus (residue 245) from residue 5 (chain A), 7 (chain B), or 9 (chain C) with the exception of four residues (98–101) in a surface loop in chain C. The CPIII-Long:C1C2 structure is isomorphous with the CPIII-His:C1C2 structure, but nearly half of CUB2 could not be modeled due to disorder (missing residues: 133–144, 164–175, 195–208, 217–227). Also, no electron density was visible for CPIII-Long for the extended region N-terminal to residues 5 (chain A), 9 (chain B), and 10 (chain C). As otherwise the two crystal structures are very similar (root-mean-square deviation [RMSD] 0.49 Å for 882 Cα atoms), the following description and figures are based on the more complete CPIII-His:C1C2 structure.

**Table 1:** Data Collection and Refinement Statistics

	CPIII-His:C1C2	CPIII-Long:C1C2
**Data Collection**		

Wavelength (Å)	0.920	0.920
Resolution range (Å)	79.4–2.70 (2.77–2.70)	68.8–2.78 (2.85–2.78)
Space group	*P*2_1_2_1_2_1_	*P*2_1_2_1_2_1_
Unit cell dimensions		
*a*, *b*, *c* (Å)	89.09, 144.12, 158.74	88.88, 143.65, 156.71
α, β, γ (°)	90, 90, 90	90, 90, 90
Unique reflections	56,733	46,452
Multiplicity	13.1 (13.7)	4.8 (5.1)
Completeness (%)	99.9 (99.9)	91.4 (94.9)
Mean *I*/σ(*I*)	17.1 (1.2)	13.1 (1.3)
CC_1/2_	0.998 (0.743)	0.997 (0.634)
*R*_merge_	0.087 (1.94)	0.066 (0.983)

**Refinement**		

Protein atoms	7,361	6,904
Solvent ions	5 Ca^2+^, 2 Cl^−^, 3 citrate	5 Ca^2+^, 3 citrate
*R*_work_	0.226	0.247
*R*_free_	0.258	0.271
RMSD bonds (Å)	0.003	0.004
RMSD angles (°)	0.74	0.71
Ramachandran plot		
Favored (%)	95.0	93.1
Allowed (%)	4.8	6.6
Outliers (%)	0.2	0.4

### Structure of the Complex

A side view of the CPIII:C1C2 complex (Figure 1B) shows the characteristic stalk, base, and petal regions of the CPIII trimer (Bourhis et al., 2012a) as well as C1C2, which is bound mostly to the stalk region, as previously suggested from low-resolution SAXS data (Bourhis et al., 2013). Importantly, when viewed down the axis of the CPIII trimer (Figure 1C), the high-resolution structure extends these previous observations to show that C1C2 binds to chains A and B of CPIII, with no binding to chain C. As shown by footprint mapping, the interactions of CUB1 and CUB2 with CPIII span the full length of the stalk region (Figure S1A).

**Figure 2:**
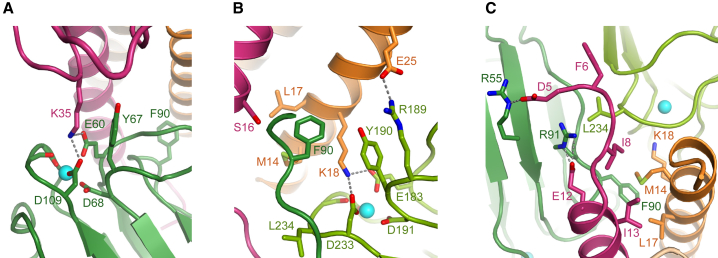
Close-ups of the Main Interactions in the Complex (A) Interaction of Lys35 (chain A) with acidic residues in the region of the Ca^2+^ binding site in CUB1. Note the stabilizing role of Tyr67 (CUB1). (B) Interaction of Lys18 (chain B) with acidic residues in the region of the Ca^2+^ binding site in CUB2. Note the stabilizing role of Tyr190 (CUB2). Also shown is the salt bridge between Glu25 (chain B) and Arg189 (CUB2), as well as Phe90 (CUB1) buried in its hydrophobic pocket. (C) View down the axis of the CPIII coiled coil showing the N terminus of chain A at the CUB1:CUB2 interface. Leu234 (CUB2) makes hydrophobic contacts with Phe6 and Ile8 (chain A), while Arg55 (CUB1) interacts with Asp5 (chain A). Also shown is the salt bridge between Glu12 (chain A) and Arg91 (CUB1). See also Figures S2 and S3.

Close-up views of the complex (Figures 2A and 2B) reveal more details of the interactions. Lys35 in the base region of chain A (CPIII) forms salt bridges with Glu60 and Asp109 in CUB1. These acidic residues are also involved in coordination of a Ca^2+^ ion in CUB1 (see also Figure S2). In addition, Lys18 in chain B (CPIII) forms salt bridges with Glu183 and Asp233 in CUB2, which again are involved in Ca^2+^ coordination. Interactions involving Lys18 and Lys35 with Ca^2+^ binding sites in C1C2 were previously predicted based on site-directed mutagenesis (Blanc et al., 2007, Bourhis et al., 2013), although it was not known at the time that these involved two different chains of CPIII. In addition to Lys18 and Lys35, the structure reveals hitherto unknown roles for the highly conserved residues Glu12 and Glu25 in stabilizing the complex with C1C2. Thus, Glu12 in chain A (CPIII) forms a salt bridge with Arg91 (CUB1; Figure 2C), while Glu25 in chain B (CPIII) forms a salt bridge with Arg189 (CUB2; Figure 2B). Furthermore, the highly conserved Tyr67 in CUB1 and its equivalent in CUB2, Tyr190 (see sequence alignment in Blanc et al., 2007), stabilize Lys35 in chain A and Lys18 in chain B of CPIII, respectively, via hydrophobic interactions with their hydrocarbon chains (Figures 2A, 2B, and S2).

Except for the first few residues in chain A (see below), all of the C1C2 interacting residues in chain A of CPIII bind solely to CUB1. In addition to Glu12 and Lys35 involved in salt bridges, these comprise Ser16 and Ser19/Gln23 which form van der Waals and hydrophobic interactions with Thr89/Phe90 and Leu61, respectively, of CUB1. Phe90 (CUB1) was previously shown to be essential for PCPE-1-enhancing activity (Blanc et al., 2007). As well as its interactions with chain A, Phe90 interacts with chain B via Met14, Leu17, and Lys18 (Figure 2B), thus forming a hydrophobic core in the center of the CPIII:C1C2 complex. Apart from Phe90 (CUB1), chain B interacts mainly with CUB2, including Lys18 and Glu25 involved in salt bridges, an H bond between Asp11 and Val236 (CUB2), Met14 and Thr15 interfacing with Ser235 and Val236 (CUB2), and His48 with Tyr187 (CUB2). Finally, Asn21 (chain B) interfaces with Leu61 (CUB1) and Tyr187/Arg189 (CUB2), and Glu25 (chain B) interfaces with Pro63 (CUB1). Altogether, the total buried surface area between C1C2 and CPIII is 1,935 Å^2^, made up of 838 Å^2^ between chain A and CUB1, 257 Å^2^ between chain A and CUB2, 316 Å^2^ between chain B and CUB1, and 524 Å^2^ between chain B and CUB2.

In addition to the interactions between CPIII and C1C2, there are interactions between CUB1 and CUB2, accounting for a total buried surface area of 794 Å^2^. In CUB1, these involve residues projecting from one edge of the jelly-roll structure in the region of Pro63, Gln81, Phe90, and Ala93. These interact with residues projecting from one face of CUB2 in the region of Tyr187/Arg189, Asp202/Asp203, Tyr190, and Leu234, respectively (Figure S1B). With the exception of Gln81 (CUB1) which forms an H bond with Asp203 (CUB2), all these contacts are van der Waals or hydrophobic interactions. This edge-face interaction is also characterized by an approximately 45° rotation between the long axes of the two CUB domains (Figure 1D). When compared with the relatively elongated low-resolution structure of full-length PCPE-1 in solution (Bernocco et al., 2003, Kronenberg et al., 2009), the ability of CUB1 and CUB2 to interact in this way in the complex confirms that there is considerable flexibility in the inter-domain linker.

### Toward the Mechanism of Action of PCPE-1

A particularly interesting finding was the interaction between the N-terminal residues of CPIII chain A with C1C2. For chains B and C, the visible N-terminal regions begin at Lys7 and Asn9, respectively, and do not interact with C1C2. In contrast, the visible N-terminal region of chain A begins with residue Asp5 and appears to be “pulled” toward C1C2 (Figure 1D), where it is accommodated in the cleft between its two CUB domains (Figure 2C). In particular, Asp5 (chain A) forms a salt bridge with Arg55 (CUB1) and its carbonyl oxygen forms a hydrogen bond with Arg91 (CUB1), which itself forms a salt bridge with Glu12 (chain A). In addition, Phe6 (chain A) is buried in an apolar trench involving Pro94, Pro157, Ile159, and Leu234 in C1C2 (Figure S3), where Leu234 also makes hydrophobic contacts with Ile8 (chain A) (Figure 2C). Similar observations were made with the CPIII-Long:C1C2 structure.

These interactions involving the N-terminal sequence of CPIII suggest a possible molecular mechanism by which PCPE-1 enhances the release of the C-propeptides from fibrillar procollagens. Binding of C1C2 to one of the three chains of the C-propeptide trimer might unravel the procollagen molecule in the region of the proteolytic cleavage site. Since the active site of the BTPs is too small to accommodate all three procollagen chains (MacSweeney et al., 2008), this would isolate chain A allowing it to enter the active site, thus accelerating the action of BTPs. To test this hypothesis, we carried out site-directed mutagenesis of residues in C1C2 that interact with the “pulled” N terminus of chain A (CPIII), followed by enzyme assays and surface plasmon resonance (SPR) to measure their effects on enhancing activity and substrate binding. For these experiments we used mini-procollagen III as a substrate, which is similar to CPIII-Long but includes a much longer region of the C-terminal triple-helical region (Moali et al., 2005).

**Figure 3:**
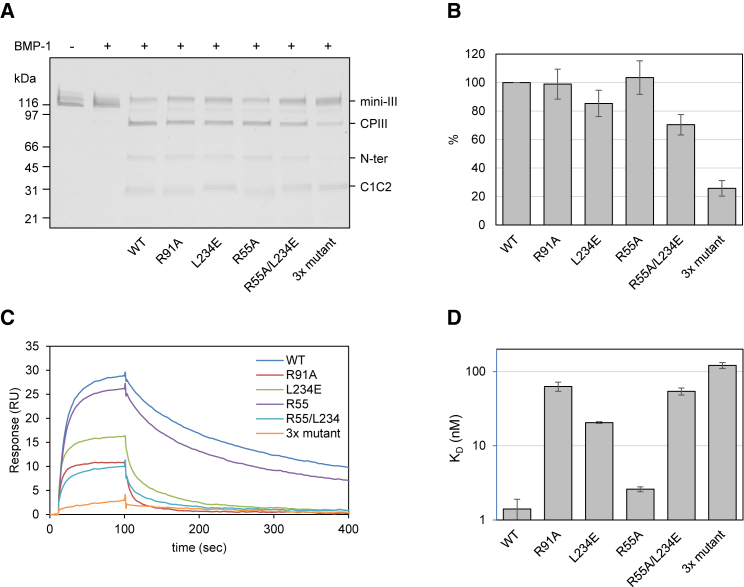
Enhancing Activities and Binding Affinities of C1C2 Mutants (A) Representative SDS-PAGE data showing the enhancement of BMP-1 proteolytic activity, using a mini-procollagen III substrate (mini-III), by C1C2 (wild-type [WT] and mutants), as revealed by the increased amounts of CPIII and N-terminal fragment (N-ter) released. Non-reducing conditions, Coomassie blue staining. (B) Quantitation of enhancing activity measured by the percentage of product released for each mutant normalized to wild-type C1C2. Data show means ± SD from at least 3 independent experiments. (C) Representative SPR data showing the interaction of 48 nM C1C2 (wild-type and mutants) injected over immobilized mini-procollagen III (240 RU) at a continuous flow rate of 50 μL/min, temperature 25°C. (D) Dissociation constants (nM) obtained from the SPR data using a 1:1 binding model with error bars (±SD) based on at least 3 experiments. Note logarithmic scale. See also Figure S4.

The effects of the following C1C2 mutations were studied (Figure 3A): Arg91Ala, Leu234Glu, Arg55Ala, Arg55Ala/Leu234Glu, and Arg55Ala/Arg91Ala/Leu234Glu. Neither Arg55Ala nor Arg91Ala affected enhancing activity (Figure 3B) relative to wild-type C1C2. For Leu234Glu there was a decrease of 15% and with the double mutant (Arg55Ala/Leu234Glu) the decrease was 30%. With the triple mutant (Arg55Ala/Arg91Ala/Leu234Glu), enhancing activity fell by 75%.

Previous studies have shown that the enhancing activity of PCPE-1 follows closely its affinity for procollagen. For example, alanine mutations at Lys18 or Lys35 in CPIII, or at Asp68 or Asp191 in PCPE-1, were sufficient to raise dissociation constants beyond measurable levels (Blanc et al., 2007, Bourhis et al., 2013). We therefore used SPR to measure the binding affinities of the C1C2 mutants presented here. As shown in Figures 3C and 3D, mutations Arg91Ala and Leu234Glu increased dissociation constants about 50- and 20-fold, respectively. In contrast, little change in affinity was seen with the Arg55Ala mutation. For the double mutant (Arg55Ala/Leu234Glu), again there was an approximately 50-fold increase in dissociation constant (*K*_D_), while for the triple mutation (Arg55Ala/Arg91Ala/Leu234Glu) the increase was over 100-fold. In all cases, however, dissociation constants could be measured, showing the effects of these mutations to be subtler than those investigated in the past (see Discussion). Although best fits where obtained using the “heterogeneous ligand” model (as already published for wild-type C1C2 [Vadon-Le Goff et al., 2011]), values calculated using the 1:1 model are displayed in Figure 3 for an easier comparison between mutants. Full SPR and kinetics data are shown in Figure S4.

**Figure 4:**
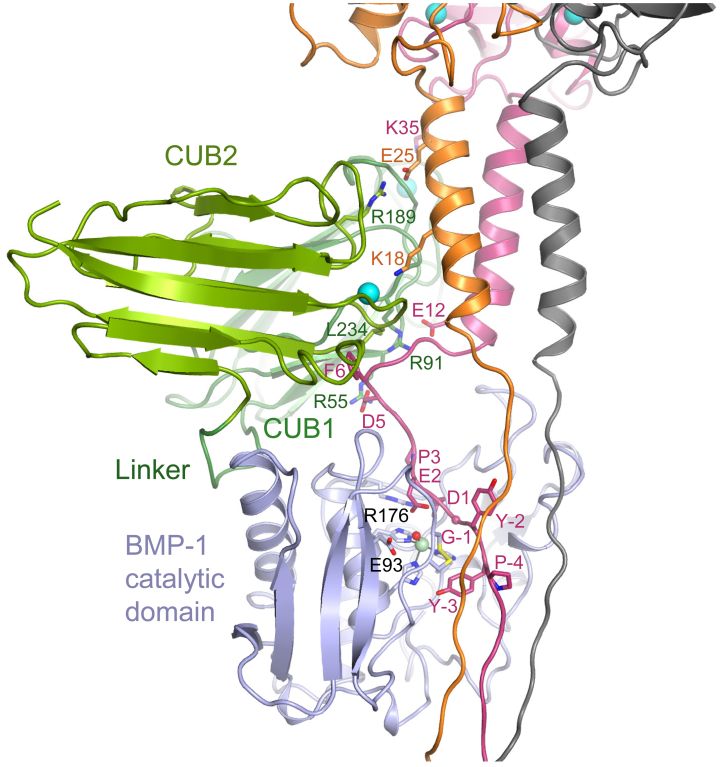
Molecular Modeling of the N-Terminal Extensions of CPIII and the Interactions of the BMP-1 Catalytic Domain Beginning with the structure of the CPIII-His:C1C2 complex, modeling extends chains A, B, and C of CPIII toward the C-telopeptide region. Chain A enters the active site of the BMP-1 catalytic domain where the P1′ residue, Asp1, interacts with Arg176 in the S1′ pocket, in close proximity to the essential Glu93 and the catalytic water molecule bound to the active-site zinc. Also shown are Cys64 and Cys65, which form a vicinal disulfide bond in the region of Tyr-2 and Tyr-3 in chain A. The negative sign indicates residues on the non-prime side of the cleavage site. See also Figure S5.

Finally, to test whether the “pulled” chain might be accessible to cleavage by BTPs, we modeled the interaction of the BMP-1 catalytic domain with the procollagen:C1C2 complex (Figure S5). To do so, since there is limited structural information available on the C-telopeptide region that connects the C-propeptide to the triple-helical region of the procollagen molecule (Jones and Miller, 1987, Liu et al., 1993, Malone and Veis, 2004), we clamped this region at each end using the CPIII:C1C2 structures reported here and that of the C-terminal region of collagen III (Boudko et al., 2008). For BMP-1, we used the crystal structure of the catalytic domain (MacSweeney et al., 2008), which packed close to C1C2 in the complex, burying an additional surface area of 785 Å^2^. In this way it was possible to extend the N-terminal region of chain A into the active-site cleft of BMP-1 (Figure 4) with Asp1 bound to Arg176 in the S1′ pocket and the scissile bond positioned for nucleophilic attack by a zinc-bound water molecule polarized by the essential Glu93. It also placed two tyrosine residues on the N-terminal side of the cleavage site close to the vicinal disulfides, in accordance with the putative role of the latter in hydrophobic interactions (Richardson et al., 2017).

## Discussion

We report here the high-resolution structures of the CUB domains of PCPE-1. Both domains share the same structural features as the subset of Ca^2+^-binding CUB domains found in complement proteases, neuropilins, cubilin, and tumor necrosis factor-stimulated gene 6 protein (TSG-6) (Appleton et al., 2007, Andersen et al., 2010, Gaboriaud et al., 2011, Janssen et al., 2012, Briggs et al., 2015). These features include the two 4-stranded β sheets, three conserved acidic residues, and a tyrosine residue involved in Ca^2+^ coordination. In addition, we found that Tyr67 and Tyr190 stabilize the interactions involving Lys35 (chain A) and Lys18 (chain B), respectively. These stabilizing tyrosines are also conserved in the CUB domains of neuropilins, complement proteases, and BTPs, but not those of cubilin (Figure S2) or TSG-6.

We also compared the structure of CPIII in the complex with that of CPIII alone (Bourhis et al., 2012a). For the trimer, an RMSD value of 1.25 Å (669 α carbons) was obtained. When comparing individual chains, however, the RMSD for chain A (0.91 Å) was higher than for chains B and C (0.63 Å and 0.68 Å, respectively). Close inspection shows a distortion of chain A on complex formation, in the base and stalk regions, as well as changes in the relative orientations of the chains. We also note that the asymmetry previously reported for CPIII alone, where one end of helix 4 in one of the three chains is distorted, is unchanged when bound to C1C2. The residues involved are Leu138 and Leu139. In addition, the same chain has altered side-chain conformations for Arg39, Arg42, and Phe46, with or without C1C2 bound. All these residues are involved in chain-chain interactions, and the asymmetry is found in chain A. This feature might specifically direct C1C2 binding to the AB face of CPIII.

The structures reported here, where one molecule of C1C2 binds to chains A and B of the CPIII trimer, immediately explain why maximum enhancing activity requires a 1:1 molar ratio of interactants. With only chain C available, binding of additional molecules of C1C2 is impossible. Concerning binding affinity, previous studies have shown that decreases in PCPE-1 enhancing activity, following mutations in Lys18 and Lys35 and their binding partners in C1C2, are mirrored by increased dissociation constants (Blanc et al., 2007, Bourhis et al., 2013). This was not always the case in the results reported here. As expected, the Arg55Ala mutation in C1C2 had no effect on enhancing activity and there was no significant increase in dissociation constant. In contrast, the Arg91Ala mutation had no effect on enhancing activity despite a 50-fold increase in dissociation constant, while a similar increase in *K*_D_ was accompanied by a detectable reduction in enhancing activity for the double mutant Arg55Ala/Leu234Glu. To explain these observations, we propose that enhancing activity requires tight binding of C1C2 to the stalk/base region of CPIII and additional binding to the N-terminal region in order to isolate chain A. Both these interactions contribute to overall affinity. For the same increase in *K*_D_, mutations in C1C2 affecting interactions with the stalk region of CPIII (such as Arg91Ala) will have less effect on enhancing activity than mutations affecting interactions with the N-terminal region (such as Arg55Ala/Leu234Glu).

In the CPIII-Long:C1C2 structure, no electron density was visible for CPIII-Long in the region encompassing the BMP-1 cleavage site, the C-telopeptide, the cystine knot and the short triple helix and the N-terminal His tag (Figures 1A and S5). Yet the interactions involving the N-terminal residues Asp5 to Ile8 in chain A were identical to those in the CPIII-His:C1C2 structure. This shows that the presence of the N-terminal His tag in CPIII-His did not change the interactions of chain A with C1C2. It also shows that the C-telopeptide region is mostly unstructured, as previously suggested by modeling and nuclear magnetic resonance studies (Jones and Miller, 1987, Liu et al., 1993, Malone and Veis, 2004). There is just one modeling study of the C-telopeptides when all three chains are anchored at each end by the C-propeptides and the collagen triple helix (Malone et al., 2005), and this was for procollagen I. A feature of this region is the transition from the collagen triple helix, in which the three chains are staggered axially by 2.86 Å, to the coiled-coil region in the C-propeptides, where all three chains are in register (Beck and Brodsky, 1998). This inevitably leads to a looser structure for the more C-terminal so-called trailing strand in the triple helix, which corresponds to the looped chain A in the model (Figure 4). It also led to kinks in chains B and C in the crosslinking region in the model that are not present in chain A (Figure S5).

The model was built assuming that BMP-1 cleaves the chain that is “pulled” into the C1C2 interface. An alternative possibility is that once chain A has been teased out by C1C2, chains B or C would become more available for proteolysis. Energetically, however, the model shown here is preferred, given that this also includes interactions between C1C2 and BMP-1, which would help direct the enzyme to chain A. This is consistent with the observed increased binding of an inactive form of BMP-1 to mini-procollagen III when this substrate is pre-bound with C1C2 (Bourhis et al., 2013), albeit that the binding of C1C2 to BMP-1 alone is weak (Bekhouche et al., 2010), suggesting cooperativity in binding of BMP-1 to both C1C2 and CPIII. Presumably once the first chain has been cleaved, chains B and C should become more exposed and therefore more susceptible to proteolysis, perhaps involving redocking of PCPE-1 to the propeptide regions of these chains. This system is similar to the cleavage of collagen molecules by matrix metalloproteinases (MMPs), where again the active site of the protease is too small to accommodate all three chains of the substrate (Bertini et al., 2012, Manka et al., 2012, Stura et al., 2013, Van Doren, 2015, Prior et al., 2016). In this case it seems that the MMP hemopexin and catalytic domains contribute to shifting the equilibrium state of the cleavage-site region toward a locally unfolded triple helix. It is likely that a similar scenario occurs with the procollagen system, where PCPE-1 plays the role of the hemopexin domain. But the procollagen system is even more complex as BTPs themselves include a number of non-catalytic CUB and EGF domains involved in substrate recognition and PCPE-1 activity (Hartigan et al., 2003). The precise roles of these domains remain to be elucidated.

## STAR★Methods

### Key Resources Table

**Table:** 

REAGENT or RESOURCE	SOURCE	IDENTIFIER
**Bacterial and Virus Strains**		

*E*. *coli* XL10-Gold	Agilent Technologies	Cat #200521
*E*. *coli* DH5α	Thermo Fisher Scientific	Cat# 18265017

**Chemicals**, **Peptides**, **and Recombinant Proteins**		

Polyethylenimine, linear, MW 25000	Polysciences	Cat #23966-1
Wizard 3 crystallization screen, conditions 1 and 16	Molecular Dimensions	Cat #MD15-W3-T

**Deposited Data**		

Crystal structure of CPIII-His	(Bourhis et al., 2012a)	PDB: 4AE2
Crystal structure of CPIII-His	(Bourhis et al., 2012a)	PDB: 4AK3
Crystal structure of the CUB_C domain of TSG-6	(Briggs et al., 2015)	PDB: 2WNO
Crystal structure of the BMP-1 catalytic domain	(MacSweeney et al., 2008)	PDB: 3EDH
Crystal structure of the collagen III C-terminal triple helix	(Boudko et al., 2008)	PDB: 3DMW
Crystal structure of CPIII-His:C1C2	This paper	PDB: 6FZV
Crystal structure of CPIII-Long:C1C2	This paper	PDB: 6FZW

**Experimental Models**: **Cell Lines**		

FreeStyle™ 293F cells	Thermo Fisher Scientific	Cat #R79007

**Oligonucleotides**		

PCR primers for cloning into pCEP-Pu: F: 5′- TCGCTAGCCCAGACCCCCAACTACG CCAGACCC -3′ R: 5′- TAGGCGGCCGCCTTGGTACCGAGAG TGCCCCGCG -3′	Sigma-Aldrich	N/A
PCR primers for site-directed mutagenesis: R55A: F: 5′-TGTGTCCCTCTCATTCGCAGTCTTCGA CCTGGAG-3′ R: 5′-CTCCAGGTCGAAGACTGCGAATGAGA GGGACACA-3' R91A: For: 5′-TTGTGGGACCTTCGCGCCTGCGCCCCTA-3′ Rev: 5′-TAGGGGCGCAGGCGCGAAGGTCCCACAA-3′ L234E: F: 5′-CGTCCAGTTCGTCTCAGATGAGAGTGT CACCGCTGATGG-3′ R: 5′-CCATCAGCGGTGACACTCTCATCTGAG ACGAACTGGACG-3′	Sigma-Aldrich	N/A

**Recombinant DNA**		

pHLsec-CPIII-His	(Bourhis et al., 2012b)	N/A
pHLsec-CPIII-Long	(Bourhis et al., 2013)	N/A
pHLsec-CUB1CUB2	(Bourhis et al., 2013)	N/A
pCEP4-mini-procollagen-III	(Moali et al., 2005)	N/A
pCEP4-BMP-1	(Blanc et al., 2007)	N/A
Modified pCEP-Pu vector	(Kohfeldt et al., 1997)	N/A

**Software and Algorithms**		

XDS	(Kabsch, 2010)	http://xds.mpimf-heidelberg.mpg.de/
XIA2	(Winter et al., 2013)	https://xia2.github.io/index.html
POINTLESS	(Evans, 2011)	http://www.ccp4.ac.uk/html/pointless.html
AIMLESS	(Evans and Murshudov, 2013)	http://www.ccp4.ac.uk/html/aimless.html
CTRUNCATE	(Winn et al., 2011)	http://www.ccp4.ac.uk/html/ctruncate.html
PHASER	(McCoy et al., 2007)	http://www.phaser.cimr.cam.ac.uk/index.php/Phaser_Crystallographic_Software
PHENIX	(Adams et al., 2010)	https://www.phenix-online.org/
COOT	(Emsley and Cowtan, 2004)	https://www2.mrc-lmb.cam.ac.uk/personal/pemsley/coot/
PyMol	Schrödinger	https:/pymol.org
ImageQuant	GE Healthcare	Cat #29291749
Biacore T200 software v3.0	GE Healthcare	Cat #29-1486-95
LSQKAB	(Kabsch, 1976)	http://www.ccp4.ac.uk/html/lsqkab.html
YASARA	(Krieger and Vriend, 2014)	http://www.yasara.org/

### Contact for Reagents and Resources Sharing

Further information and requests for resources and reagents should be directed to and will be fulfilled by the Lead Contact, David Hulmes (david.hulmes@ibcp.fr).

### Experimental Model and Subject Details

#### Cell Culture

Human (female) FreeStyle™ HEK293-F cells (Thermo Fisher Scientific) were grown in FreeStyle™ 293 Expression Medium at 37°C with 8% CO_2_.

### Method Details

#### Protein Expression and Purification

All coding sequences were obtained by PCR amplification from cDNAs. CPIII-His and CPIII-Long with N-terminal fused His-tags cloned into the pHLsec vector have been described (Bourhis et al., 2012b, Bourhis et al., 2013). Their numbering starts at the P1′ position in the BTP cleavage site (Figure 1A) with negative numbers used on the N-terminal side. The C1C2 fragment of PCPE-1 spans residues 1 to 252 of the mature protein (i.e. after removal of the 25-residue signal sequence, UniProt Q15113). For structural studies, C1C2 with a C-terminal fused His-tag cloned into the pHLsec vector was used (Bourhis et al., 2013). For the preparation of C1C2 mutants (and wild-type control), cDNA coding for C1C2 was first cloned into a modified pCEP-Pu vector (Kohfeldt et al., 1997). In the secreted protein, a vector-derived APLA sequence is present at the N-terminus, and a fused His-tag (AAAHHHHHH) is present at the C-terminus. The C1C2 point mutations were introduced using QuikChange II XL (Agilent Technologies).

Proteins were expressed using the FreeStyle™ 293 Expression System (Thermo Fisher Scientific) following the manufacturer's protocols. Briefly, 293F cells were grown in a shaking incubator at 37°C with 8% CO_2_ in serum-free 293 Expression Medium to a cell density of 10^6^ cells/ml. Transfections were performed using linear polyethylenimine (PEI; MW 25,000; Polysciences) and a DNA:PEI ratio of 1:3 (w/w). Conditioned medium containing the secreted proteins was collected 5 days after transfection. Filtered medium was adjusted by addition of 1 M Na-Hepes pH 7.5 to a final concentration of 20 mM then loaded onto a 5-mL HisTrap Excel column (GE Healthcare) pre-equilibrated in 20 mM Na-Hepes pH 7.5, 2.5 mM CaCl_2_ and 0.15 M NaCl (CPIII-His or CPIII-Long) or 0.5 M NaCl (C1C2-His), using an ÄKTA Pure chromatography system (GE Healthcare). Following a washing step with buffer containing 10 mM imidazole, proteins were eluted with buffer containing 200 mM imidazole. Fractions containing protein were concentrated using Vivaspin centrifugal devices (Sartorius) to a final concentration of ∼5 mg/ml then further purified on a Superdex 200 10/300 Increase column (GE Healthcare) using 20 mM Na-Hepes pH 7.5, 2.5 mM CaCl_2_ and 500 mM NaCl as running buffer.

Purified CPIII-His or CPIII-Long was complexed with purified C1C2 by overnight incubation at room temperature at a 9:6 mass ratio (i.e. molar ratio 1:2). The resulting protein complexes where then separated from excess C1C2 on a Superdex 200 10/300 Increase column (GE Healthcare) using 20 mM Na-Hepes pH 7.5, 2.5 mM CaCl_2_ and 500 mM NaCl as running buffer. Fractions containing the complex were then pooled and concentrated using Vivaspin centrifugal devices (Sartorius), using 20 mM Na-Hepes pH 7.5, 2.5 mM CaCl_2_ to lower the NaCl concentration to 0.18M, to a final protein concentration of ∼20 mg/ml.

#### Crystallization and Structure Determination

Screening was done at 20°C by the sitting-drop vapor diffusion method using 96-well plates (Greiner) and a range of commercial screens. A mosquito Nanolitre Robot (TTP Labtech) was used to set up 200 nl drops. The initial crystals of CPIII-His:C1C2 were obtained using a protein concentration of 20 mg/ml and 0.2 M potassium citrate, 20% PEG3350 as precipitant, and for CPIII-Long:C1C2 using a protein concentration of 20 mg/mL and 0.2 M ammonium citrate, 20% PEG3350 (Wizard Classic screen, Molecular Dimensions). Larger crystals were grown in 2 μl hanging drops using 10% to 25% PEG3350, and the best crystals were obtained at 16% PEG3350 (CPIII-His:C1C2) and 18 % PEG3350 (CPIII-Long-CUBCUB2). Crystals were flash-frozen in liquid nitrogen using a reservoir solution supplemented with 20% ethylene glycol as cryoprotectant.

Diffraction data were collected at 100 K on beamline I04-1 at the Diamond Light Source (Oxfordshire, UK). The data were processed using XDS (Kabsch, 2010) and programs of the CCP4 suite, including AIMLESS (Evans and Murshudov, 2013), CTRUNCATE (Winn et al., 2011) and POINTLESS (Evans, 2011) as implemented in the XIA2 pipeline (Winter et al., 2013). CC1/2 (Karplus and Diederichs, 2012) in AIMLESS was used to determine the resolution limit. Phases were determined by molecular replacement using PHASER (McCoy et al., 2007) as implemented in the PHENIX suite (Adams et al., 2010). Search models were derived from the crystal structure of CPIII (Bourhis et al., 2012a) (PDB: 4AE2, 4AK3) and the CUB_C domain of TSG-6 (Briggs et al., 2015) (PDB: 2WNO). Manual rebuilding and refinement were done using COOT (Emsley and Cowtan, 2004) and PHENIX. Strong spherical electron density features at two positions were interpreted as chloride ions based on their chemical environments and the high concentration of chloride in the crystallization buffer. Figures were generated using PyMOL (www.pymol.org).

#### Enzyme Kinetics and Binding Studies

To measure the enhancing activity of wild-type and mutant forms of C1C2, 400 nM mini-procollagen III (Moali et al., 2005) was incubated for 1h at 37°C with 2.5 nM BMP-1 (Blanc et al., 2007) in the absence and presence of 500 nM C1C2 (wild-type or mutants) in 20 mM Na-Hepes pH 7.4, 150 mM NaCl, 5 mM CaCl_2_, 0.02% Brij-35, in a total reaction volume of 30 μL. These conditions were chosen to give approximately 10% conversion of the mini-procollagen substrate in 1h in the absence of C1C2. Reactions were stopped on ice then 5X Laemmli sample buffer was added and the samples prepared for SDS-PAGE analysis (non-reducing conditions) using BioRad 4-20% gradient gels and staining with Coomassie Blue. Enhancing activity is expressed as the percentage of product released for each mutant normalized to wild-type CUBCUB2.

Surface plasmon resonance experiments were performed using a Biacore T200 (GE Healthcare) at the Protein Science Facility of the UMS3444 (Lyon). Immobilization of mini-procollagen III (two different surfaces), regeneration of sensor chips and analysis of kinetics were as previously described (Blanc et al., 2007, Kronenberg et al., 2009). Sensorgrams were recorded at 25°C using 10 mM Na-Hepes pH 7.4, 0.15 M NaCl, 5 mM CaCl_2_, 0.05 % P20 as running buffer. Model fitting was carried out using the 1:1 binding and heterogeneous ligand models in the Biacore T200 software v3.0.

#### Structure Analysis and Molecular Modeling

Structural alignments were done using LSQKAB (Kabsch, 1976). Molecular modeling was performed using YASARA Structure (Krieger and Vriend, 2014) with the Amber 14 force field (Hornak et al., 2006). Missing intra-chains were build using the YASARA Buildloop command (Canutescu and Dunbrack, 2003) before being minimized under constraints. Prior to minimization, hydrogen bonding network optimization was performed using the YASARA OptHydAll command (Krieger et al., 2012). To build the model, the first step was to build the region of chain A containing the Gly-Asp cleavage site (Figure 1A) into the BMP-1 active site. This required a small movement, compared to the crystal structure, of the vicinal disulfide bond in BMP-1 (Cys64-Cys65; PDB: 3EDH) in accordance with its known flexibility (MacSweeney et al., 2008) (Video S1). We then linked this model to the crystal structure by building the short intervening sequence in chain A, which, due to packing constraints, gave rise to interactions between C1C2 and BMP-1. To build the remainder of the C-telopeptides, all three chains were built as loops beginning at the C-terminus of the triple helix then linked to the corresponding chains. For this, two constraints were used for the energy minimization: (i) a cystine knot structure corresponding to model 3 of Boudko et al. (2008) (PDB: 3DMW) at the triple-helix/C-telopeptide junction, and (ii) inter-chain salt bridges involving the conserved Glu-Lys sequence involved in collagen cross-linking (Yamauchi and Sricholpech, 2012), as suggested for the C-telopeptides of collagen I (Malone and Veis, 2004). We also built the C1C2 linker region that was not visible in the electron density maps. All steric clashes were avoided at each step.

Video S1. Molecular Dynamics Simulation of the BMP-1 Catalytic Domain, Related to Figure 4 Highlighted in green is the relatively mobile vicinal disulfide bond that partially occludes the active site, as well as the catalytic zinc in magenta and Arg176 in the S1′ pocket in blue.

### Quantification and Statistical Analysis

For the enzyme kinetics studies, gels were scanned and peak intensities quantified using ImageQuant (GE Healthcare Life Sciences). Data for enzyme kinetics and surface plasmon resonance are shown as means ± SD based on at least 3 experiments.

### Data and Software Availability

The accession numbers for the structures of CPIII-His:C1C2 and CPIII-Long:C1C2 reported in this paper are PDB: 6FZV and PDB: 6FZW, respectively. The coordinates of the model shown in Figures 4 and S5 are available on request.

## Supplemental Information

Document S1. Figures S1–S5

Document S2. Article plus Supplemental Information

## References

[bib1] Adams P.D., Afonine P.V., Bunkoczi G., Chen V.B., Davis I.W., Echols N., Headd J.J., Hung L.W., Kapral G.J., Grosse-Kunstleve R.W. (2010). PHENIX: a comprehensive Python-based system for macromolecular structure solution. Acta Crystallogr. D Biol. Crystallogr..

[bib2] Adar R., Kessler E., Goldberg B. (1986). Evidence for a protein that enhances the activity of type I procollagen C-proteinase. Coll. Relat. Res..

[bib3] Andersen C.B.F., Madsen M., Storm T., Moestrup S.K., Andersen G.R. (2010). Structural basis for receptor recognition of vitamin-B(12)-intrinsic factor complexes. Nature.

[bib4] Appleton B.A., Wu P., Maloney J., Yin J., Liang W.C., Stawicki S., Mortara K., Bowman K.K., Elliott J.M., Desmarais W. (2007). Structural studies of neuropilin/antibody complexes provide insights into semaphorin and VEGF binding. EMBO J..

[bib5] Beck K., Brodsky B. (1998). Supercoiled protein motifs: the collagen triple-helix and the alpha-helical coiled coil. J. Struct. Biol..

[bib6] Bekhouche M., Kronenberg D., Vadon-Le Goff S., Bijakowski C., Lim N.H., Font B., Kessler E., Colige A., Nagase H., Murphy G. (2010). Role of the NTR domain of procollagen C-proteinase enhancer-1 in the control of metalloproteinase activity. J. Biol. Chem..

[bib7] Bernocco S., Steiglitz B.M., Svergun D.I., Petoukhov M.V., Ruggiero F., Ricard-Blum S., Ebel C., Geourjon C., Deleage G., Font B. (2003). Low resolution structure determination shows procollagen C-proteinase enhancer to be an elongated multi-domain glycoprotein. J. Biol. Chem..

[bib8] Berry R., Jowitt T.A., Ferrand J., Roessle M., Grossmann J.G., Canty-Laird E.G., Kammerer R.A., Kadler K.E., Baldock C. (2009). Role of dimerization and substrate exclusion in the regulation of bone morphogenetic protein-1 and mammalian tolloid. Proc. Nat. Acad. Sci. USA.

[bib9] Bertini I., Fragai M., Luchinat C., Melikian M., Toccafondi M., Lauer J.L., Fields G.B. (2012). Structural basis for matrix metalloproteinase 1-catalyzed collagenolysis. J. Am. Chem. Soc..

[bib10] Blanc G., Font B., Eichenberger D., Moreau C., Ricard-Blum S., Hulmes D.J.S., Moali C. (2007). Insights into how CUB domains can exert specific functions while sharing a common fold: conserved and specific features of the CUB1 domain contribute to the molecular basis of procollagen C-proteinase enhancer-1 activity. J. Biol. Chem..

[bib11] Boudko S.P., Engel J., Okuyama K., Mizuno K., Bachinger H.P., Schumacher M.A. (2008). Crystal structure of human type III collagen Gly991-Gly1032 cystine knot-containing peptide shows both 7/2 and 10/3 triple helical symmetries. J. Biol. Chem..

[bib12] Bourhis J.M., Mariano N., Zhao Y., Harlos K., Exposito J.Y., Jones E.Y., Moali C., Aghajari N., Hulmes D.J.S. (2012). Structural basis of fibrillar collagen trimerization and related genetic disorders. Nat. Struct. Mol. Biol..

[bib13] Bourhis J.M., Mariano N., Zhao Y., Walter T.S., El Omari K., Delolme F., Moali C., Hulmes D.J.S., Aghajari N. (2012). Production and crystallization of the C-propeptide trimer from human procollagen III. Acta Crystallogr. Sect. F Struct. Biol. Cryst. Commun..

[bib14] Bourhis J.M., Vadon-Le Goff S., Afrache H., Mariano N., Kronenberg D., Thielens N.M., Moali C., Hulmes D.J.S. (2013). Procollagen C-proteinase enhancer grasps the stalk of the C-propeptide trimer to boost collagen precursor maturation. Proc. Nat. Acad. Sci. USA.

[bib15] Briggs D.C., Birchenough H.L., Ali T., Rugg M.S., Waltho J.P., Ievoli E., Jowitt T.A., Enghild J.J., Richter R.P., Salustri A. (2015). Metal ion-dependent heavy chain transfer activity of TSG-6 mediates assembly of the cumulus-oocyte matrix. J. Biol. Chem..

[bib16] Canutescu A.A., Dunbrack R.L. (2003). Cyclic coordinate descent: a robotics algorithm for protein loop closure. Protein Sci..

[bib17] Emsley P., Cowtan K. (2004). Coot: model-building tools for molecular graphics. Acta Crystallogr. D Biol. Crystallogr..

[bib18] Evans P.R. (2011). An introduction to data reduction: space-group determination, scaling and intensity statistics. Acta Crystallogr. D Biol. Crystallogr..

[bib19] Evans P.R., Murshudov G.N. (2013). How good are my data and what is the resolution?. Acta Crystallogr. D Biol. Crystallogr..

[bib20] Gaboriaud C., Gregory-Pauron L., Teillet F., Thielens N.M., Bally I., Arlaud G.J. (2011). Structure and properties of the Ca(2+)-binding CUB domain, a widespread ligand-recognition unit involved in major biological functions. Biochem. J..

[bib21] Hartigan N., Garrigue-Antar L., Kadler K.E. (2003). Bone morphogenetic protein-1 (BMP-1). Identification of the minimal domain structure for procollagen C-proteinase activity. J. Biol. Chem..

[bib22] Hornak V., Abel R., Okur A., Strockbine B., Roitberg A., Simmerling C. (2006). Comparison of multiple Amber force fields and development of improved protein backbone parameters. Proteins.

[bib23] Janssen B.J., Malinauskas T., Weir G.A., Cader M.Z., Siebold C., Jones E.Y. (2012). Neuropilins lock secreted semaphorins onto plexins in a ternary signaling complex. Nat. Struct. Mol. Biol..

[bib24] Jones E.Y., Miller A. (1987). Structural models for the N- and C-terminal telopeptide regions of interstitial collagens. Biopolymers.

[bib25] Kabsch W. (1976). A solution for the best rotation to relate two sets of vectors. Acta Crystallogr..

[bib26] Kabsch W. (2010). XDS. Acta Crystallogr. D Biol. Crystallogr..

[bib27] Kadler K.E., Hojima Y., Prockop D.J. (1987). Assembly of collagen fibrils de novo by cleavage of the type I pC- collagen with procollagen C-proteinase. Assay of critical concentration demonstrates that collagen self-assembly is a classical example of an entropy-driven process. J. Biol. Chem..

[bib28] Karplus P.A., Diederichs K. (2012). Linking crystallographic model and data quality. Science.

[bib29] Kohfeldt E., Maurer P., Vannahme C., Timpl R. (1997). Properties of the extracellular calcium binding module of the proteoglycan testican. FEBS Lett..

[bib30] Krieger E., Dunbrack R.L., Hooft R.W., Krieger B. (2012). Assignment of protonation states in proteins and ligands: combining pKa prediction with hydrogen bonding network optimization. Methods Mol. Biol..

[bib31] Krieger E., Vriend G. (2014). YASARA View—molecular graphics for all devices—from smartphones to workstations. Bioinformatics.

[bib32] Kronenberg D., Vadon-Le Goff S., Bourhis J.M., Font B., Eichenberger D., Hulmes D.J.S., Moali C. (2009). Strong cooperativity and loose geometry between CUB domains are the basis for procollagen C-proteinase enhancer activity. J. Biol. Chem..

[bib33] Liu X.H., Otter A., Scott P.G., Cann J.R., Kotovych G. (1993). Conformational analysis of the type II and type III collagen α-1 chain C-telopeptides by H-1 NMR and circular dichroism spectroscopy. J. Biomol. Struct. Dyn..

[bib34] MacSweeney A., Gil-Parrado S., Vinzenz D., Bernardi A., Hein A., Bodendorf U., Erbel P., Logel C., Gerhartz B. (2008). Structural basis for the substrate specificity of bone morphogenetic protein 1/tolloid-like metalloproteases. J. Mol. Biol..

[bib35] Malone J.P., Alvares K., Veis A. (2005). Structure and assembly of the heterotrimeric and homotrimeric C-propeptides of type I collagen: significance of the α2(I) chain. Biochemistry.

[bib36] Malone J.P., Veis A. (2004). Heterotrimeric type I collagen C-telopeptide conformation as docked to its helix receptor. Biochemistry.

[bib37] Manka S.W., Carafoli F., Visse R., Bihan D., Raynal N., Farndale R.W., Murphy G., Enghild J.J., Hohenester E., Nagase H. (2012). Structural insights into triple-helical collagen cleavage by matrix metalloproteinase 1. Proc. Nat. Acad. Sci. USA.

[bib38] McCoy A.J., Grosse-Kunstleve R.W., Adams P.D., Winn M.D., Storoni L.C., Read R.J. (2007). Phaser crystallographic software. J. Appl. Crystallogr..

[bib39] Mehal W.Z., Iredale J., Friedman S.L. (2011). Scraping fibrosis: expressway to the core of fibrosis. Nat. Med..

[bib40] Moali C., Font B., Ruggiero F., Eichenberger D., Rousselle P., Francois V., Oldberg A., Bruckner-Tuderman L., Hulmes D.J.S. (2005). Substrate-specific modulation of a multisubstrate proteinase. C-terminal processing of fibrillar procollagens is the only BMP-1-dependent activity to be enhanced by PCPE-1. J. Biol. Chem..

[bib41] Moschcovich L., Bernocco S., Font B., Rivkin H., Eichenberger D., Chejanovsky N., Hulmes D.J.S., Kessler E. (2001). Folding and activity of recombinant human procollagen C-proteinase enhancer. Eur. J. Biochem..

[bib42] Moschcovich L., Kessler E. (2016). Data comparing the kinetics of procollagen type I processing by bone morphogenetic protein 1 (BMP-1) with and without procollagen C-proteinase enhancer 1 (PCPE-1). Data Brief.

[bib43] Petropoulou V., Garrigue-Antar L., Kadler K.E. (2005). Identification of the minimal domain structure of bone morphogenetic protein-1 (BMP-1) for chordinase activity: chordinase activity is not enhanced by procollagen C-proteinase enhancer-1 (PCPE-1). J. Biol. Chem..

[bib44] Prior S.H., Byrne T.S., Tokmina-Roszyk D., Fields G.B., Van Doren S.R. (2016). Path to collagenolysis: collagen V triple-helix model bound productively and in encounters by matrix metalloproteinase-12. J. Biol. Chem..

[bib45] Ricard-Blum S. (2011). The collagen family. Cold Spring Harb. Perspect. Biol..

[bib46] Richardson J.S., Videau L.L., Williams C.J., Richardson D.C. (2017). Broad analysis of vicinal disulfides: occurrences, conformations with cis or with trans peptides, and functional roles including sugar binding. J. Mol. Biol..

[bib47] Rockey D.C., Bell P.D., Hill J.A. (2015). Fibrosis—a common pathway to organ injury and failure. N. Engl. J. Med..

[bib48] Sharma U., Carrique L., Vadon-Le Goff S., Mariano N., Georges R.N., Delolme F., Koivunen P., Myllyharju J., Moali C., Aghajari N. (2017). Structural basis of homo- and heterotrimerization of collagen I. Nat. Commun..

[bib49] Stura E.A., Visse R., Cuniasse P., Dive V., Nagase H. (2013). Crystal structure of full-length human collagenase 3 (MMP-13) with peptides in the active site defines exosites in the catalytic domain. FASEB J..

[bib50] Vadon-Le Goff S., Hulmes D.J.S., Moali C. (2015). BMP-1/tolloid-like proteinases synchronize matrix assembly with growth factor activation to promote morphogenesis and tissue remodeling. Matrix Biol..

[bib51] Vadon-Le Goff S., Kronenberg D., Bourhis J.M., Bijakowski C., Raynal N., Ruggiero F., Farndale R.W., Stöcker W., Hulmes D.J.S., Moali C. (2011). Procollagen C-proteinase enhancer stimulates procollagen processing by binding to the C-propeptide only. J. Biol. Chem..

[bib52] Van Doren S.R. (2015). Matrix metalloproteinase interactions with collagen and elastin. Matrix Biol..

[bib53] Wermter C., Howel M., Hintze V., Bombosch B., Aufenvenne K., Yiallouros I., Stöcker W. (2007). The protease domain of procollagen C-proteinase (BMP1) lacks substrate selectivity, which is conferred by non-proteolytic domains. Biol. Chem..

[bib54] Winn M.D., Ballard C.C., Cowtan K.D., Dodson E.J., Emsley P., Evans P.R., Keegan R.M., Krissinel E.B., Leslie A.G., McCoy A. (2011). Overview of the CCP4 suite and current developments. Acta Crystallogr. D Biol. Crystallogr..

[bib55] Winter G., Lobley C.M., Prince S.M. (2013). Decision making in xia2. Acta Crystallogr. D Biol. Crystallogr..

[bib56] Yamauchi M., Sricholpech M. (2012). Lysine post-translational modifications of collagen. Essays Biochem..

