# Supplementary material for: Structural Basis for the Acceleration of Procollagen Processing by Procollagen C-Proteinase Enhancer-1
Source: Structure. 2018 Oct 2;26(10):1384–1392.e3. doi: 10.1016/j.str.2018.06.011 (PMC6372009; doi:10.1016/j.str.2018.06.011)
Supplement: Document S1. Figures S1–S5 [file mmc1.pdf]

Structure, Volume *26*

Supplemental Information

Structural Basis for the Acceleration of Procollagen Processing by Procollagen C-Proteinase Enhancer-1

David Pulido, Urvashi Sharma, Sandrine Vadon-Le Goff, Sadaf-Ahmahni Hussain, Sarah Cordes, Natacha Mariano, Emmanuel Bettler, Catherine Moali, Nushin Aghajari, Erhard Hohenester, and David J.S. Hulmes

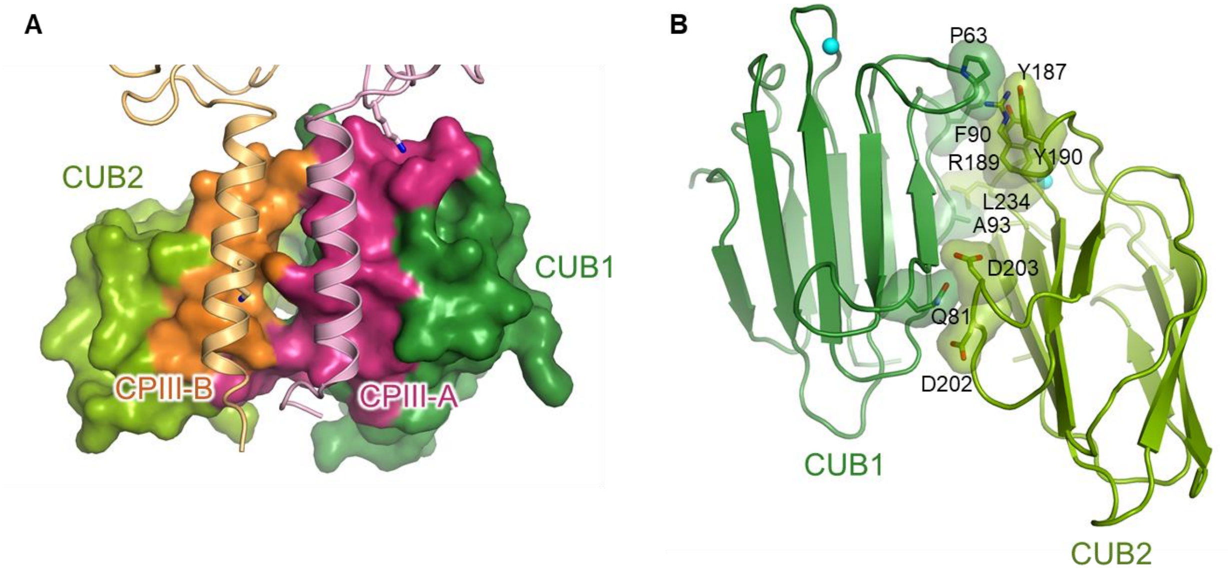

Figure S1. Related to Figure 1. CPIII binding interfaces and inter-domain interactions of C1C2. (**A**) C1C2 solvent-accessible surface buried upon complex formation with CPIII. Interaction sites for chains A and B are shown as color-coded foot prints on C1C2. For clarity, chain C has been removed. (**B**) Cartoon representation of C1C2 in the CPIII-His:C1C2 complex. Residues involved in key inter-domain interactions are shown as sticks, and Ca^{2+} ions in cyan.

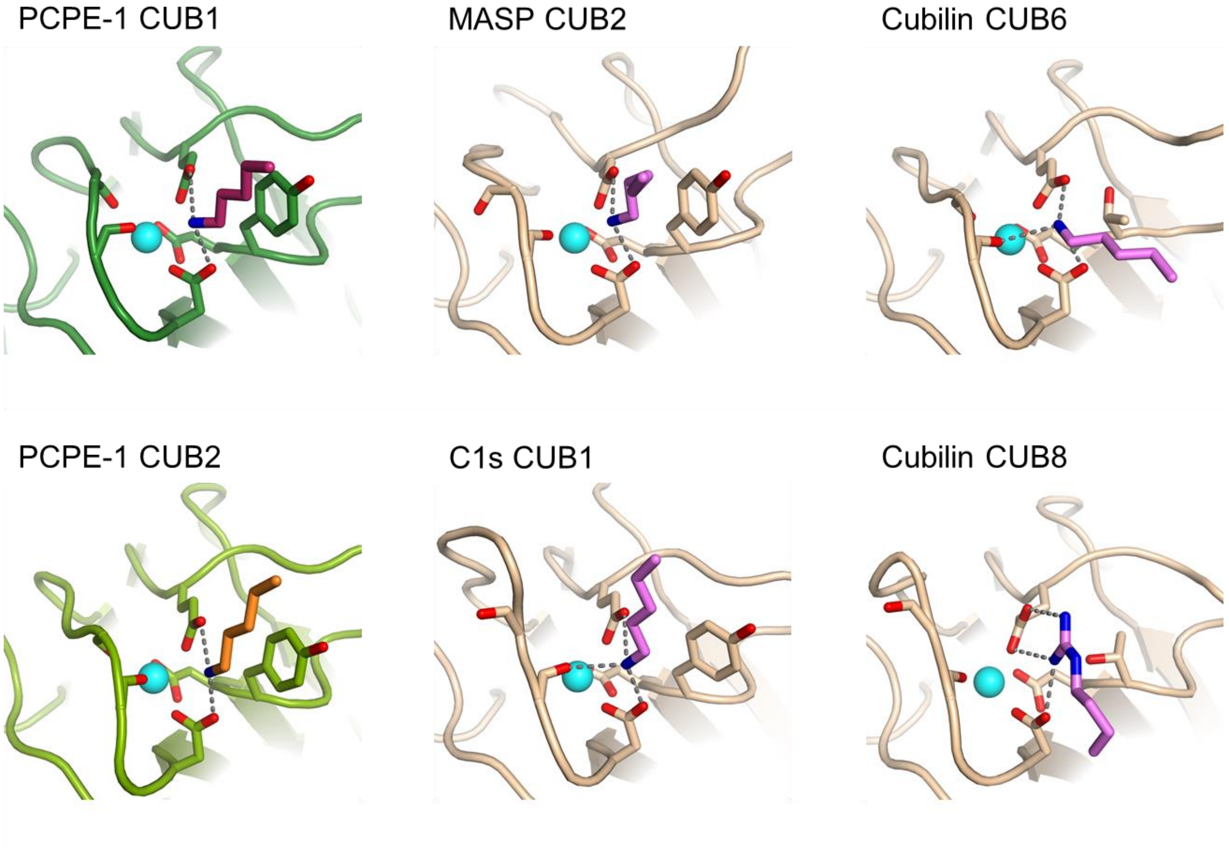

Figure S2 - Related to Figure 2. Structural comparison of lysine interactions with Ca^{2+} binding regions in different protein complexes. Note the stabilizing role for adjacent tyrosine residues in the CUB domains of PCPE-1 and the complement proteases MASP-1 and C1s.

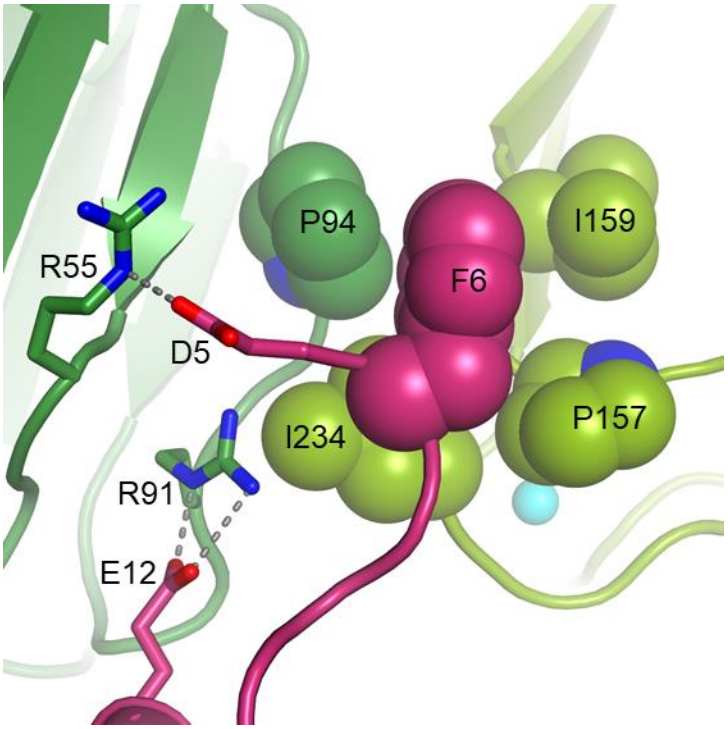

Figure S3. Related to Figure 2. Space-filling view of the interactions of Phe6 (chain A). Phe6 is buried in an apolar trench involving Pro94, Pro157, Ile159 and Leu234 in C1C2.

A

1:1 binding

Sample	K_D (nM)	k_a (1/Ms)	k_d (1/s)	Chi² (RU²)
**WT**	1.4 ± 0.5	4.2 ± 1.7 x 10^6	0.005 ± 0.0009	0.3 - 2.9
**R91A**	63 ± 9	2.0 ± 0.3 x 10^6	0.128 ± 0.030	0.2 - 0.5
**L234E**	20.5 ± 0.6	1.4 ± 0.4 x 10^6	0.029 ± 0.008	1.4 - 2.5
**R55A**	2.6 ± 0.2	2.9 ± 0.9 x 10^6	0.008 ± 0.002	0.4 - 0.9
**R55A/L234E**	54 ± 6	5.8 ± 0.5 x 10^5	0.032 ± 0.002	1.3 - 2.7
**triple mutant**	121 ± 11	4.1 ± 0.2 x 10^4	0.005 ± 0.0004	1.5 - 1.6

B

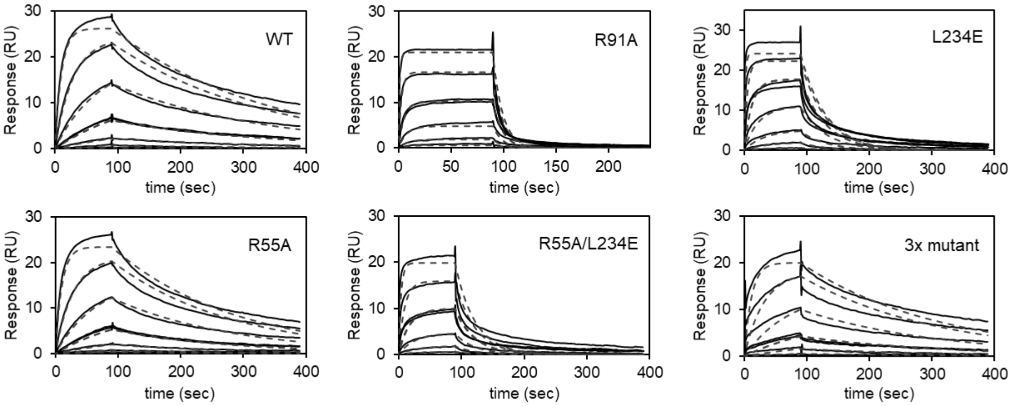

C

heterogeneous ligand

Sample	K_{D1} (nM)	k_{a1} (1/Ms)	k_{d1} (1/s)	K_{D2} (nM)	k_{a2} (1/Ms)	k_{d2} (1/s)	R_{max1} (%)	R_{max2} (%)	Chi² (RU²)
**C1C2 WT**	0.82 ± 0.02	2.46 ± 0.01 x10^7	0.0202 ± 0.0004	2.38 ± 0.02	1.70 ± 0.01 X10^6	0.0040 ± 0.0000	27 ± 6	73 ± 6	0.20
**C1C2 R91A**	3.6 ± 0.2	2.91 ± 0.11 x10^6	0.0105 ± 0.0002	85 ± 1	2.16 ± 0.02 X10^6	0.1829 ± 0.0016	6 ± 3	94 ± 3	0.20
**C1C2 L234E**	7.8 ± 0.1	1.00 ± 0.01 x10^6	0.0078 ± 0.0001	36 ± 1	3.44 ± 0.04 X10^6	0.1245 ± 0.0013	30 ± 7	70 ± 7	0.50
**C1C2 R55A**	1.65 ± 0.03	1.84 ± 0.01 x10^6	0.0030 ± 0.0000	5.3 ± 0.1	4.00 ± 0.05 X10^6	0.0213 ± 0.0003	40 ± 4	60 ± 4	0.26
**C1C2 R55A/L234E**	31 ± 1	1.79 ± 0.02 x10^5	0.0055 ± 0.0001	64 ± 3	1.87± 0.06 X10^6	0.1196 ± 0.0033	71 ±1	29 ± 1	0.31
**triple mutant**	131 ± 3	2.32 ± 0.03 x10^4	0.0030 ± 0.0000	198 ± 21	5.65 ± 0.05 X10^5	0.1117 ± 0.0072	73	27	1.00

D

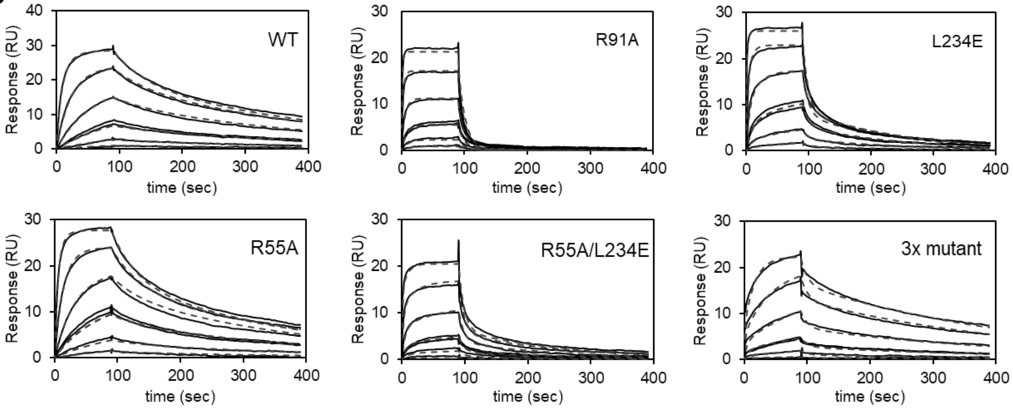

Figure S4. Related to Figure 3. Full kinetic data and sensorgrams from SPR analysis. (**A**) Kinetic and equilibrium dissociation constants determined by SPR after injection of C1C2 over immobilized mini-procollagen III (240-280 RU), using the 1:1 binding model. Three-fold serial dilutions of C1C2 were injected: 0-80 nM for both wild-type (WT) and the R55A mutant; 0-420 nM for the R91A, L234E and R55A/L234E mutants and 0-2.1µM for the triple mutant. Flow rate 50 µl/min, temperature 25°C. Means ± SD for at least 3 experiments (two different surfaces). Biacore T200 software v3.0. (**B**) Representative sensorgrams corresponding to (A) with fits shown by the dotted lines. (**C**) Same data as (A) but fitted using the heterogeneous ligand model. (**D**) Representative sensorgrams corresponding to (C) with fits shown by the dotted lines.

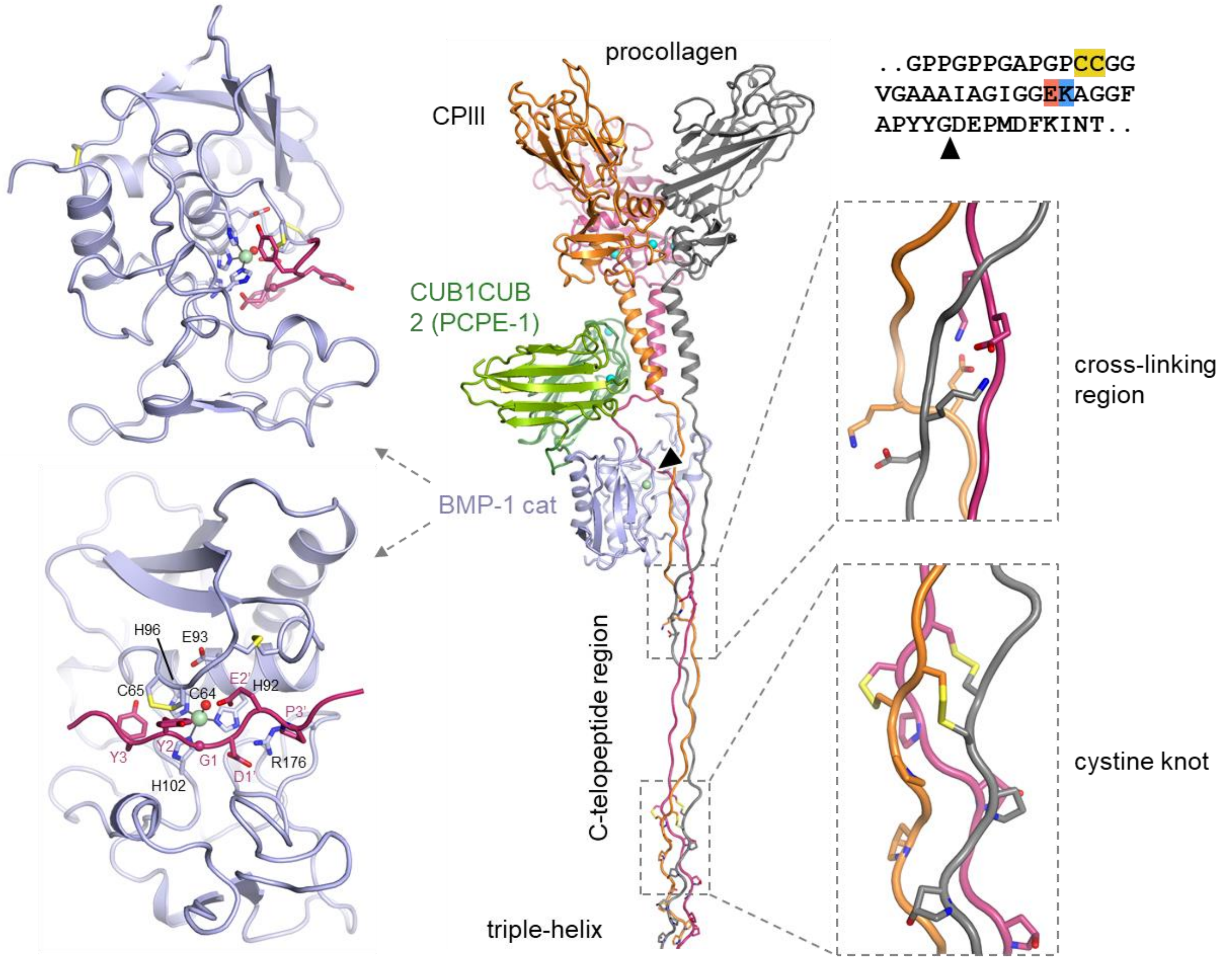

Figure S5. Related to Figure 4. Modeling of the CPIII:C1C2:BMP-1 complex. Different views of the complex including the full-size model with chains A, B and C of CPIII continuing through the C-telopeptide region to the collagen triple helix. The BTP cleavage site is indicated by the arrowhead. Also shown are zooms of (i) the BMP-1 catalytic domain (orthogonal views) with part of chain A buried in the active site (primed residues are C-terminal to the cleavage site), (ii) the conserved residues Glu-Lys in the intermolecular cross-linking region, and (iii) the inter-chain cystine knot at the C-telopeptide/triple helix junction. Locations of the vicinal cysteines, residues Glu-Lys in the cross-linking region and the BTP cleavage site are also indicated in the sequence above which connects the C-terminal triple-helical region to the N-terminus of the C-propeptide.
